# Evaluation of the role of *FMR1* CGG repeat allele in Parkinson’s disease from the Chinese population

**DOI:** 10.3389/fnagi.2023.1234027

**Published:** 2023-07-31

**Authors:** Juan Chen, Yuwen Zhao, Xun Zhou, Jin Xue, Qiao Xiao, Hongxu Pan, Xiaoxia Zhou, Yaqin Xiang, Jian Li, Liping Zhu, Zhou Zhou, Yang Yang, Qian Xu, Qiying Sun, Xinxiang Yan, Jieqiong Tan, Jinchen Li, Jifeng Guo, Ranhui Duan, Beisha Tang, Qiao Yu, Zhenhua Liu

**Affiliations:** ^1^Department of Neurology, Xiangya Hospital, Central South University, Changsha, Hunan, China; ^2^Bioinformatics Center, National Clinical Research Center for Geriatric Disorders, Xiangya Hospital, Central South University, Changsha, Hunan, China; ^3^Department of Geriatrics, Xiangya Hospital, Central South University, Changsha, Hunan, China; ^4^Centre for Medical Genetics and Hunan Key Laboratory of Medical Genetics, School of Life Sciences, Central South University, Changsha, Hunan, China; ^5^Department of Nuclear Medicine, Xiangya Hospital, Central South University, Changsha, China; ^6^Department of Radiology, Xiangya Hospital, Central South University, Changsha, China; ^7^Key Laboratory of Hunan Province in Neurodegenerative Disorders, Central South University, Changsha, Hunan, China

**Keywords:** *FMR1* gene, CGG repeat, Parkinson’s disease, premutation, gray zone

## Abstract

**Objective:**

There is controversial evidence that *FMR1* premutation or “gray zone” (GZ) allele (small CGG expansion, 45–54 repeats) was associated with Parkinson’s disease (PD). We aimed to explore further the association between *FMR1* CGG repeat expansions and PD in a large sample of Chinese origin.

**Methods:**

We included a cohort of 2,362 PD patients and 1,072 controls from the Parkinson’s Disease and Movement Disorders Multicenter Database and Collaborative Network in China (PD-MDCNC) in this study and conducted repeat-primed polymerase chain reaction (RP-PCR) for the size of *FMR1* CGG repeat expansions.

**Results:**

Two PD patients were detected with *FMR1* premutation (61 and 56 repeats), and the other eleven PD patients were detected with the GZ allele of *FMR1* CGG repeat expansions. Those thirteen PD patients responded well to levodopa and were diagnosed with clinically established PD. Specifically, one female PD patient with GZ allele was also found with premature ovarian failure. However, compared to healthy controls, we found no significant enrichment of GZ allele carriers in PD patients or other subgroups of PD cases, including the subgroups of female, male, early-onset, and late-onset PD patients. Furthermore, we did not find any correlation between the *FMR1* gene CGG repeat sizes and age at onset of PD.

**Conclusion:**

It suggested that *FMR1* premutation was related to PD, but the GZ allele of *FMR1* CGG repeat expansions was not significantly enriched in PD cases of Chinese origin. Further larger multiple ethnic studies are needed to determine further the role of the *FMR1* GZ allele in PD.

## 1. Introduction

Parkinson’s disease (PD) is characterized by motor and non-motor symptoms, including bradykinesia, resting tremors, and rigidity (Bloem et al., 2021; Zhou et al., 2022). As one of the most common neurodegenerative diseases, PD is considered to be caused by genetic and environmental factors and aging. Specifically, more than 20 monogenic Mendelian inheritance genes have been identified over the last decades (Blauwendraat et al., 2020; Liu et al., 2022). Several monogenic causes, such as *SNCA* and *LRRK2* genes, may reproduce the deposition of α-synuclein in cytoplasmic Lewy bodies, a pathological hallmark of PD. Furthermore, over 90 loci were found to be the risk factor of PD by genome-wide association study (GWAS) or genotype analyses (Nalls et al., 2019; Pan et al., 2023). Among them, heterozygous glucosidases (*GBA*) gene variants were found to be the high-risk factor with an over 10-fold increased risk of PD and an earlier age at onset (Pan et al., 2023).

However, only a small proportion of idiopathic PD was confirmed to be linked to known PD disease-causing genes (Zhao et al., 2020). For example, from our previous study, only about 7.8% of idiopathic PD were identified as pathogenic or likely pathogenic of known PD disease-causing genes; most of them were single nucleotide variations, small indels. Recently, short tandem repeats (STRs), especially GGC repeats, were considered to be a risk factor for PD, including intermediate-length GGC repeat expansions of *NOTCH2NLC* (Tian et al., 2019; Huang et al., 2022), intermediate-length GGGGCC repeat expansion of *C9orf72* (Cali et al., 2019), and GGC repeat expansion of *GIPC1* (Pan et al., 2022). Therefore, the potential role of *FMR1* CGG repeat expansion as a risk factor in PD is needed to further exploration.

The fragile X messenger ribonucleoprotein 1 (*FMR1*) gene is located on the X chromosome. Fragile X-associated disorders are caused by a CGG repeat expansion in the 5’ untranslated region (5’ UTR) of the *FMR1* gene, including fragile X-associated tremor/ataxia syndrome (FXTAS), of which the number of CGG is among 55–199 (premutation range) and fragile X syndrome (FXS) of which the number of CGG is over 200 (full mutation range) (Hagerman and Hagerman, 2021). Besides, millions of people are carriers of smaller expansions (gray zone) in the trinucleotide repeat of the *FMR1* gene. However, less attention has been paid to diseases that occur in patients with *FMR1* “gray zone” (GZ) expansion carriers (Hall et al., 2020). However, the number of CGG for GZ alleles is not uniform, with some studies initially suggesting 45–54 and extending it to 35–60 in other studies (Loesch et al., 2009). In this study, the GZ allele was considered to have 45–54 CGGs.

Considering that the symptoms of PD, essential tremor (ET), and spinocerebellar ataxia (SCA) overlap to some extent with those of FXTAS, previous studies have screened these patients for CGG repeat expansion of the *FMR1* gene (Cilia et al., 2009). It has been suggested that some patients with multiple system atrophy or PD may also be carriers of mutations that lead to the CGG repeat expansion of the *FMR1* gene (Hall et al., 2005). The core clinical manifestations of *FMR1* premutation alleles were cerebellar ataxia and action tremor; specifically, about one-third of those affected presented with Parkinsonian symptoms (Hagerman, 2013). Furthermore, several previous population-based datasets supported that the occurrence of the GZ allele was enriched in male or female patients with parkinsonism (Loesch et al., 2018). A recent Greek study also reported two PD patients out of 171 with PD with *FMR1* premutation (Kartanou et al., 2022).

However, existing evidence from previous studies is still controversial. For example, no significant enrichment of the GZ allele was reported in 414 patients with Parkinsonism (Toft et al., 2005). Then, a subsequent study consisting of 903 Caucasian PD or essential tremor patients showed no significant excess of GZ allele carriers in cases either (Kraff et al., 2007). Furthermore, another study, including a small sample size cohort (66 non-specified parkinsonism and 74 normal controls), reported no association between cases and controls (Reis et al., 2008). The limitation of those negative studies were relatively small size, different ethnic or geographic regions, less stringent inclusion criteria, et al. Therefore, a larger sample size and more consistent inclusion criteria were required to assess the association between idiopathic PD and controls in the Chinese population.

In this study, we presented data from a larger sample that provides statistical evidence for the important role of the *FMR1* gene CGG repeat expansions as a risk factor for PD and stimulates thinking about possible mechanisms by which these alleles may act synergistically with other PD susceptibility factors.

## 2. Materials and methods

### 2.1. Participants

A total of 2,362 PD patients from mainland China recruited from the Xiangya Hospital, Central South University, were enrolled in the Parkinson’s Disease and Movement Disorders Multicenter Database and Collaborative Network in China (PD-MDCNC)^1^ (Zhou et al., 2022). All PD participants fulfilled the Movement Disorders Society (MDS) clinical diagnostic criteria for PD (Postuma et al., 2015). Furthermore, 1,072 healthy populations without neurological diseases were recruited from the community or were the spouse of the recruited PD cases. The demographic data of those participants are shown in Table 1.

**TABLE 1:** Basic demographic characteristics of participants.

Cohorts	Entire cohort (*n* = 2362)	Female cohort (*n* = 1119)	Male cohort (*n* = 1243)	EOPD cohort (*n* = 1118)	LOPD cohort (*n* = 1244)	Control group (*n* = 1072)
*Age/age at onset (year, mean ± SD)	53.61 ± 11.62	54.06 ± 10.99	53.20 ± 12.14	43.70 ± 6.34	62.51 ± 7.25	62.95 ± 7.09
Sex (male/female)	1243/1119	0/1119	1243/0	615/503	628/616	520/552

*The age for the health controls and the age at onset for the cases; EOPD, early onset of Parkinson’s disease; LOPD, late onset of Parkinson’s disease.

This study was approved by the Ethics Committee of Xiangya Hospital (Central South University), and all subjects’ written informed consent was collected. After obtaining written informed consent, peripheral blood samples were collected from all participants. Genomic DNA was extracted from peripheral white blood cells following standard procedures.

### 2.2. Data collection and clinical examination

A comprehensive set of primary demographic data, including participants’ age, sex, family history, course of the disease, and clinical features (motor and non-motor symptoms), were collected from PD patients included in this study and entered into PD-MDCNC mentioned above. The clinical manifestations of PD were assessed by several scales mentioned in our previous study (Zhou et al., 2022). Briefly, the Unified Parkinson’s Disease Rating Scale (UPDRS) was used for motor symptoms; the Parkinson’s disease sleep scale (PDSS), the REM sleep behavior disorder questionnaire-Hong Kong (RBDQ-HK), and the Epworth Sleepiness Score (ESS) were used for sleep disturbance assessment; the 17-item Hamilton Depression Rating Scale (HAMD) was used for depression assessment; the Hyposmia Rating Scale (HRS) was used for olfactory assessment; the Mini-Mental State Examination (MMSE) was used for cognitive assessment; the Functional Constipation Diagnostic Criteria Rome III (ROME III) was used for the constipation assessment; the Scales for Outcomes in Parkinson’s disease-Autonomic (SCOPA-AUT) was used for autonomic assessment. Furthermore, an age-at-onset (AAO) of 50 years was the threshold for classifying early-onset and late-onset PD patients (patients with AAO equal to 50 are classified as early-onset PD). Then we included 1,119 EOPD patients and 1,243 LOPD patients in our study, and detailed information is shown in Table 1.

### 2.3. Genetic analysis

Repeat-primed polymerase chain reaction (RP-PCR) and fluorescent PCR were used to detect GGC repeat expansions in the *FMR1* gene, as described previously (Tian et al., 2019; Pan et al., 2022). Briefly, genomic DNA was extracted from peripheral blood using the standardized phenol-chloroform method. The number of CGG repeats of the *FMR1* gene (GenBank reference sequence and version number: GRCh37.p13) was determined by GC-rich PCR with the primers 5’AAGCCGGAGTCAGTCCGCGAGTCGAG3’ and 5’CACCAGCTCCTCCATCTTCTCTTCAG3’ using AmpliTaq Gold DNA polymerase (Applied Biosystems). The thermal cycling was as follows: denaturation at 98°C for 3 min, ten cycles of 98°C for 20 s, 65°C for 45 s, and 72°C for 3 min, followed by 22 cycles of 98°C for 20 s, 68°C for 3.5 min, and a final extension at 68°C for 10 min. The PCR product of the *FMR1* gene CGG repeat was analyzed on the ABI 3500 Genetic Analyzer (Applied Biosystems) (Gao et al., 2020).

### 2.4. Statistical analysis

All statistical analyses were performed using SPSS 26.0. The Fisher test was used for the comparison of genetic analysis between PD cases and controls. Specifically, comparing the distribution of the GZ allele of *FMR1* CGG repeat expansions between PD patients and controls was conducted after the exclusion of participants with the premutation of *FMR1* CGG repeat expansion. The linear regression and Pearson’s correlation were used to calculate the relationship between CGG repeat sizes and age at onset of PD, and the correlation coefficient *r* was determined. All *p*-values are two-tailed. The α level of 0.05 was defined as a statistically significant threshold.

## 3. Results

**FIGURE 1:**
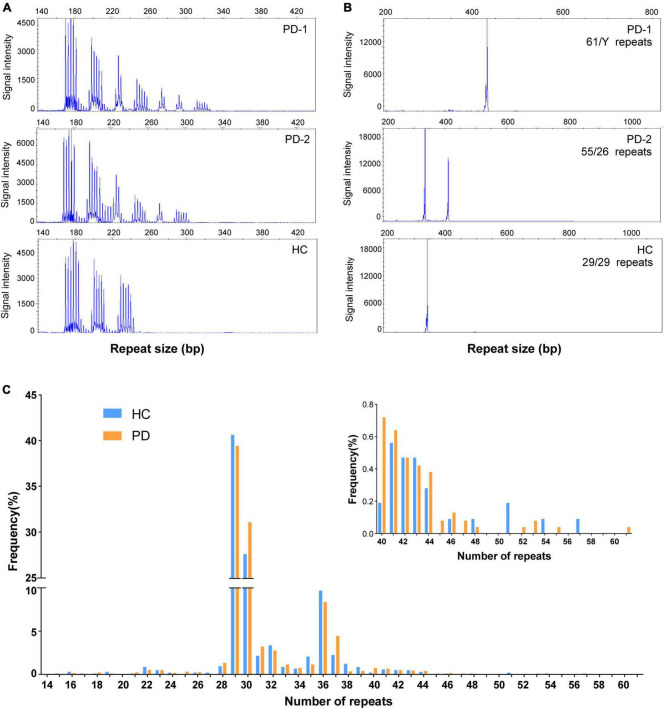
Repeat-primed polymerase chain reaction (RP-PCR) and GC-PCR validation of *FMR1* GGC repeat expansions in individuals with Parkinson’s disease and healthy control. **(A)** Electropherogram of repeat-primed PCR in PD-1/PD-2 and healthy control. All of them showed a saw-tooth pattern of the repeat expansions; **(B)** GC-PCR analysis showing the numbers of expanded GGC repeat units in the *FMR1* gene of individuals in PD-1/PD-2 and healthy control. The repeat size was labeled in each panel. **(C)** The distribution of repeat size in PD patients and healthy controls.

According to the diagnostic standard above, 2,362 PD patients were included in this study, including 241 familial PD probands (10.2%) and 2,121 sporadic PD patients (89.8%). None of them were detected with the pathogenic *FMR1* CGG repeat expansion. However, RP-PCR revealed 13 PD patients with saw-tooth waves, indicating CGG repeat expansions. Interestingly, fluorescence amplicon length analysis revealed that the premutation of *FMR1* CGG repeat expansion was detected in two patients (PD-1 and PD-2). Specifically, both patients were sporadic early-onset PD patients. Of note, GC-rich PCR showed that the repeat sizes of patients PD-1 (male) and PD-2 (female) were 61 and 55, respectively (Figures 1A, B).

**FIGURE 2:**
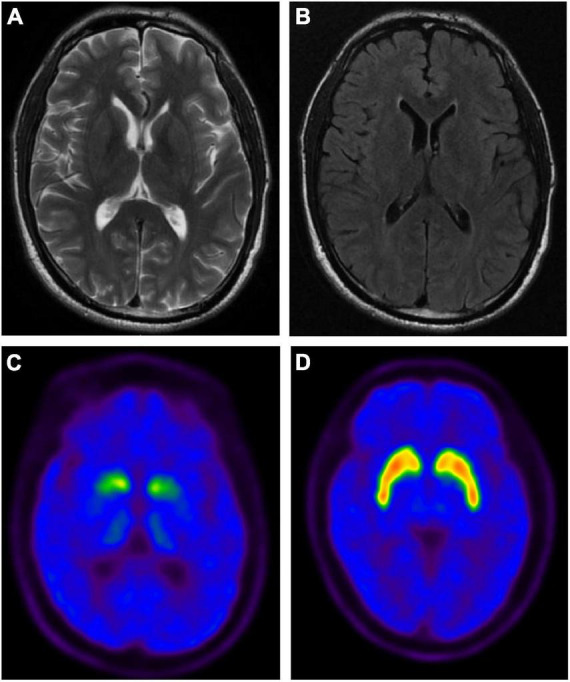
The brain magnetic resonance imaging (MRI) and positron emission tomography (PET) imaging of PD-1 patient with the premutation of *FMR1* gene CGG repeat expansion. Representative axial T2-weighted **(A)** and FLAIR **(B)** MRI images of PD-1. No abnormal signal intensities or atrophic changes are observed. Representative axial PET images of ^11^C-2β-carbomethoxy-3β-(4-fluorophenyl) tropane (^11^C-CFT) uptake in PD-1 **(C)** and healthy control (HC) **(D)**, showing the distribution of dopamine transporters in PD-1 was significantly reduced in the bilateral putamen and caudate nuclei.

The age at onset of PD-1 was 42 years. The onset symptoms of this patient were bradykinesia, followed by limb stiffness and difficulty turning over at night; then, a freezing gait appeared early, and it was easy to fall. The resting tremor, however, was relatively mild. In addition, he also showed obvious non-motor symptoms, including constipation, urinary frequency and urgency, depression, sleep disorder, and RBD, but no complaint of fluctuation of cognitive decline (MMSE scored 29 at the duration of 2 years and scored 25 at the duration of 7 years) or hallucinations. The maximum improvement rate of the L-dopa shock test was 30%. Specifically, the cranial magnetic resonance imaging (MRI) of this patient showed no abnormality, and positron emission tomography/computed tomography (PET/CT) using ^11^C-2β-carbomethoxy-3β-(4-fluorophenyl) tropane (^11^C-CFT) tracer revealed significantly impaired dopamine transmission in the striatum (Figure 2). That evidence further supported the diagnosis of PD for patient PD-1, which supported the patients with premutation of *FMR1* CGG repeat could manifest symptoms of PD. The other patient, PD-2, was onset at 44 with typical Parkinson’s disease symptoms, including resting tremor, bradykinesia, rigidity, and postural instability. This patient had been taking madopar and pramipexole to improve the symptoms and subsequently developed freezing of gait, dyskinesia, and end-of-dose phenomena. The main non-motor symptoms of this patient were depression and sleep disturbances, but there was no constipation, RBD, or hyposmia. Specifically, this patient was 50 years old with amenorrhea and no signs of premature ovarian failure. Unfortunately, this patient died in 2021 from a brain hemorrhage after the fall.

Furthermore, the GZ allele of *FMR1* CGG repeat expansion was detected in 11 patients, including eight early-onset PD patients and three late-onset PD patients. Interestingly, none of those PD patients was with PD family history. In addition, a total of 1,072 healthy populations were included in this study. Of note, none were detected with the pathogenic *FMR1* CGG repeat expansion or premutation of *FMR1* CGG repeat expansion. However, five controls were with the GZ allele of *FMR1* CGG repeat expansion.

**FIGURE 3:**
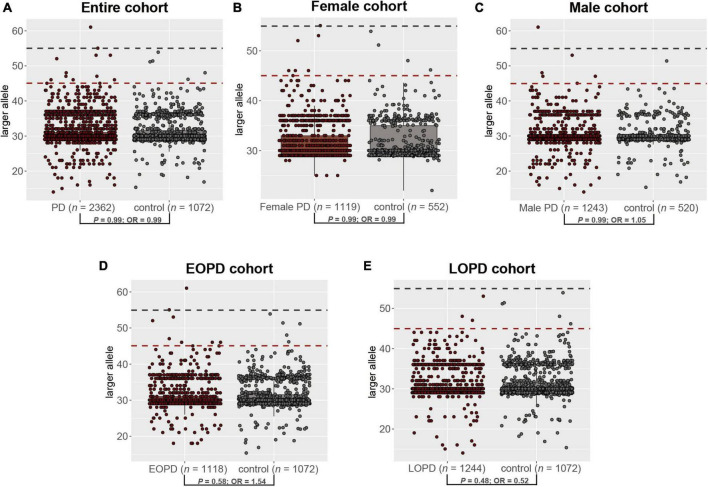
The distribution of the GZ allele of *FMR1* CGG repeat expansions between PD patients and controls in the entire cohort **(A)**, female cohort **(B)**, male cohort **(C)**, EOPD cohort **(D)**, and LOPD cohort **(E)**. PD, Parkinson’s disease; EOPD, early-onset Parkinson’s disease; LOPD, late-onset Parkinson’s disease.

The distribution of repeat length in PD patients and healthy controls is shown in Figure 1C. However, no significant difference was observed in the distribution of the GZ allele of *FMR1* CGG repeat expansions between PD patients and controls (Fisher’s exact test *P-*value = 0.99; odds ratio = 0.99; 95% confidence interval, 0.31–3.68) (Figure 3A). Then, we divided PD patients into female and male PD patients to further explore the association of the GZ allele of *FMR1* CGG repeat expansions between the gender subgroup of PD patients and controls. It showed that neither female PD patients (Fisher’s exact test *P-*value = 0.99; odds ratio = 0.99; 95% confidence interval, 0.21–6.11) nor male PD patients (Fisher’s exact test *P-*value = 0.99; odds ratio = 1.05; 95% confidence interval, 0.17–11.05) were significantly associated with GZ allele of *FMR1* CGG repeat expansions (Figures 3B, C). Next, we stratified PD patients to early-onset and late-onset patients to explore further the association of the GZ allele of *FMR1* CGG repeat expansions between the AAO subgroup of PD patients and controls. It showed that neither early-onset PD patients (Fisher’s exact test *P-*value = 0.58; odds ratio = 1.54; 95% confidence interval, 0.17–11.05) nor late-onset PD patients (Fisher’s exact test *P-*value = 0.48; odds ratio = 0.52; 95% confidence interval, 0.08–2.66) were significantly associated with GZ allele of *FMR1* CGG repeat expansions (Figures 3D, E and Supplementary Table 1).

**FIGURE 4:**
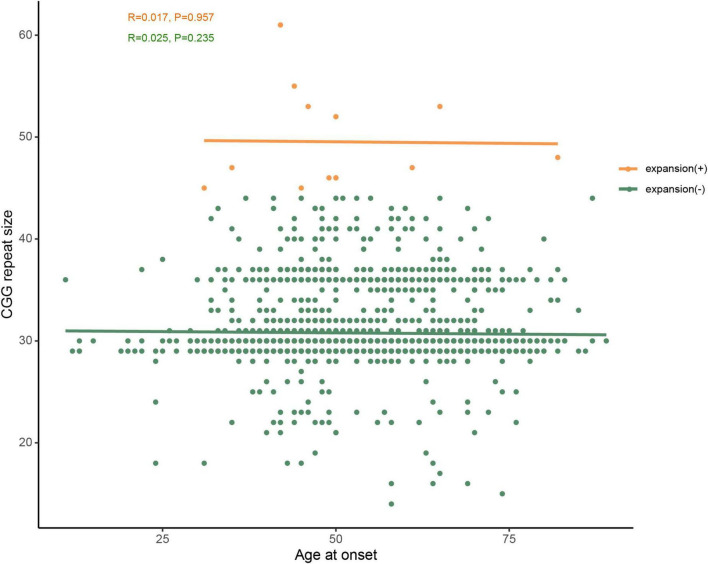
No correlation was observed between age at onset and CGG repeat size of *FMR1* gene in PD patients. Green dots represent *FMR1* gene CGG normal repeat size, whereas orange dots represent the premutation and GZ alleles of *FMR1* gene CGG repeat expansions. PD-10 and PD-11 (shown in Table 2) were at the same age onset and had the same repeat size, therefore, the two orange dots overlap.

In addition, we compared the size of *FMR1* CGG repeat between PD patients and controls, but no statistically significant difference was found (*T*-test *P-*value = 0.825; 95% confidence interval, −0.241 to 0.302), either. Furthermore, we found that the most common repeat size ranged from 28 to 37 (Figure 1C). We further investigated the association of CGG repeat size and the age at onset of PD, but no correlation was found between the CGG repeat size of the *FMR1* gene and age at onset, neither in normal repeat size (linear regression and Pearson’s correlation, *r* = 0.017, *P*-value = 0.957) nor in GZ allele (*r* = 0.025, *P*-value = 0.235) of *FMR1* gene CGG repeat (Figure 4).

The two PD patients with *FMR1* premutation (PD-1 and PD-2, one male with AAO of 42 years old and one female with AAO of 44 years old) manifested with motor symptoms, which included bradykinesia (2/2), resting tremor (2/2), rigidity (2/2), freezing gait (2/2), dyskinesia (1/2), and wearing-off (2/2), and non-motor symptoms, such as hyposmia (1/2), depression (2/2), urinary urgency (2/2), constipation (1/2), and sleep disturbance (2/2). For the patient PD-2, no premature ovarian failure symptom has been reported so far.

On the other hand, the other 11 PD patients with *FMR1* GZ allele manifested motor symptoms, which included bradykinesia (11/11), resting tremor (11/11), rigidity (11/11), freezing gait (6/11), dyskinesia (3/11), and wearing-off (11/11), and non-motor symptoms, such as cognitive dysfunction (1/11), hyposmia (9/11), depression (2/11), urinary urgency (6/11), constipation (6/11), and sleep disturbance (5/11). Among those 11 patients, there are six females and five males. There are five female and four male PD patients were followed up. Of those five female PD patients, there is only one patient (PD-5) reported the symptom of premature ovarian failure such as amenorrhea, cold intolerance, and decreased libido at age 38-year-old and the symptoms of PD at age 37-year-old, and GC-rich PCR showed that the repeat sizes of patients PD-5 were 52. However, another patient (PD-10) underwent bilateral oophorectomy for ovarian cysts at age 36-year-old, and there were no associated symptoms of premature ovarian failure at that time.

Of note, all 13 PD patients with premutation or GZ allele of *FMR1* CGG repeat expansion responded well to the levodopa therapy. Furthermore, none of them met absolute exclusion criteria or red flags for MDS clinical diagnostic criteria for PD, and all of them were diagnosed as clinically established PD (detailed information shown in Table 2).

**TABLE 2:** Clinical characteristics of patients with Parkinson’s disease harboring *FMR1* CGG expansions.

Sample	PD–1	PD–2*	PD–3*	PD–4	PD–5	PD–6	PD–7	PD–8*	PD–9	PD–10	PD–11^^^	PD–12	PD–13^^^
Repeat sizes	61	55	53	53	52	48	47	47	46	46	46	45	45
Sex	M	F	M	F	F	M	M	M	F	F	F	F	M
Age at onset, years	42	44	46	65	37	77	61	36	49	50	50	45	32
Age at exemption, years	44	50	49	68	52	81	64	45	51	51	60	52	54
Disease duration, years(base line)	2	6	3	3	15	9	3	9	2	1	10	7	22
Disease duration, years(follow–up)	7	9	11	8	9	7	8	16	8	7	17	12	34
Hoehn–Yahr stage (off)(base line)	2.5	2.5	2.0	2.0	2.0	3.0	2.0	2.0	1.0	1.0	2.5	1.0	2.5
Hoehn–Yahr stage (off)(follow–up)	4.0	/	/	3.0	2.0	4.0	2.5	/	2.0	5.0	/	2.5	/
Bradykinesia	+	+	+	+	+	+	+	+	+	+	+	+	+
Resting tremor	+	+	+	+	+	+	+	+	+	+	+	+	+
Rigidity	+	+	+	+	+	+	+	+	+	+	+	+	+
Postural instability	+	+	+	+	–	+	+	+	–	+	–	+	–
UPDRS–III (off)(base line)	29	34	21	21	6	46	31	22	9	9	41	16	26
UPDRS–III (off)(follow–up)	59	/	/	38	10	55	61	/	18	31	/	22	/
Motor subtype	PIGD	PIGD	PIGD	PIGD	PIGD	PIGD	PIGD	PIGD	TD	PIGD	PIGD	TD	TD
(Hyposmia)	+	–	+	+	–	+	+	+	+	+	+	+	–
Depression	+	+	+	–	–	–	–	–	–	–	–	+	–
Urinary urgency	+	+	+	–	+	+	+	+	–	+	–	–	–
Constipation	+	–	+	+	–	+	–	+	–	+	–	+	–
Cognitive decline	–	–	–	–	–	+	–	–	–	–	–	–	–
MMSE(base line)	29	27	28	29	29	25	29	29	27	27	27	28	29
MMSE(follow–up)	25	/	/	26	30	24	28	/	26	28	/	29	/
Sleep disturbance	+	+	+	+	–	–	+	+	–	–	–	+	–
RBD	+	–	+	+	–	–	+	–	–	+	–	–	–
Freezing gait	+	+	+	+	–	+	+	+	–	–	–	+	–
Response to levodopa	Good	Good	Good	Good	Good	Good	Good	Good	Good	Good	Good	Good	Good
Wearing off	+	+	+	+	+	+	+	+	+	+	+	+	+
Dyskinesia	–	+	+	–	–	–	+	+	–	–	–	–	–
Premature ovarian failure	NA	–	NA	–	+	NA	NA	NA	–	–	/	–	NA
Deep brain stimulation	NA	NA	NA	NA	NA	NA	NA	+	NA	NA	NA	NA	NA
PD diagnosis	EstPD	EstPDd	EstPDd	EstPDd	EstPDd	EstPDd	EstPDd	EstPDd	EstPDd	EstPDd	EstPD	EstPD	EstPD

– = absent; + = present; * = death; ^ = loss follow-up; PIGD, postural instability and gait disorders; TD, tremor dominate; PD, Parkinson’s disease; EstPD, established PD; PD diagnosis means cases were classified in accordance with the movement disorder society clinical diagnostic criteria for Parkinson’s disease.

## 4. Discussion

This study aimed to determine the role of *FMR1* gene CGG repeat expansions in PD patients from the Chinese population, and we investigated the size of *FMR1* CGG repeat expansions in a large Chinese cohort of patients with PD and healthy controls by RP-PCR and fluorescent PCR, including 2,362 patients and 1,072 controls from the Chinese mainland. We identified two patients with premutation of *FMR1* CGG repeat expansions (61 and 55 CGG repeats), whereas 11 patients and five controls with GZ allele of *FMR1* CGG repeat expansions (45–54 CGG repeats). Among those 13 PD patients, two were detected with premutation of *FMR1* CGG repeat expansions, and the other 11 patients were detected with GZ allele of *FMR1* CGG repeat expansions. However, the GZ allele of *FMR1* CGG repeat expansions was not significantly associated with PD patients or other subgroups of PD cases, including female, male, early-onset, and late-onset PD patients’ groups. Furthermore, whole-exome sequencing (WES) or whole-genome sequencing (WGS) data of those 13 PD patients (with premutation or GZ allele of *FMR1* CGG repeat expansion) did not report any pathogenic or likely pathogenic variants of known PD-associated genes (Zhao et al., 2020). Those 13 PD patients responded well to levodopa and were diagnosed with clinically established PD. Specifically, one female PD patient with GZ allele of *FMR1* CGG repeat expansions manifested with premature ovarian failure.

Previous studies also supported the role of premutation of *FMR1* CGG repeat expansion in PD. For example, an Australian study included 229 males affected with PD or other parkinsonism and 578 male controls. They found a significant excess of premutation carriers and a more than 2-fold increase in GZ allele carriers (Loesch et al., 2009). Previous studies supported the detection of *FMR1* premutation in patients with PD or Parkinsonism (Kartanou et al., 2022). However, the function of the premutation to the symptoms of PD is unclear. It might be associated with an increased risk of developing movement disorders (including PD, ET, SCA), possibly due to elevated “toxic” mRNA levels in carriers of *FMR1* premutation and GZ allele (Loesch et al., 2009). In addition, it has been shown that the toxic effect of the elevated *FMR1* transcript on blood lymphocyte levels in GZ allele carriers might lead to cellular stress and mitochondrial dysfunction, which have been verified to be associated with idiopathic PD (Loesch et al., 2011).

The GZ allele of *FMR1* CGG repeat expansions was not significantly associated with PD patients or other subgroups of PD cases. These findings were consistent with previous studies in the smaller-size cohort (Toft et al., 2005; Kraff et al., 2007; Reis et al., 2008). However, controversial results were reported in previous studies. For example, a larger Australian study containing 817 PD cases and 1,078 controls also reported an association between GZ allele carriers and increased risk for PD (Loesch et al., 2013). Another study included 2,362 participants with a prevalence of 5.2% for carriers of the *FMR1* gene GZ allele, and GZ alleles were associated with signs of Parkinsonism in men (Hall et al., 2020). These studies suggested that the *FMR1* GZ allele might contribute to the etiology of disorders associated with male Parkinsonism (Entezari et al., 2017). A similar study in China, including 360 Chinese patients with Parkinsonism (6.8%) and 295 controls, also supported this result (Zhang et al., 2012). A larger cohort included 601 female PD patients and 1,005 controls, and they found a significant excess (8.2%) of GZ allele carriers compared with 5.2% in the control. They further supported that *FMR1* gene GZ alleles are a significant risk factor for Parkinsonism in females (Loesch et al., 2018).

According to those studies, the frequency of GZ allele carriers was relatively higher in those cohorts, and the highest frequency reached 11%. Nevertheless, as we considered the range from 45 to 54 repeats as the GZ allele, the frequency was less than 1%. Considering the GZ allele range could be 40–54 repeats or 41–54 repeats (Zhang et al., 2012; Loesch et al., 2013), we also performed the analysis with those criteria. However, the highest frequency of the GZ allele in our PD cohort was still about 3%. Moreover, further analysis was conducted on the distribution of the GZ allele with different range thresholds between PD patients and controls. However, none of them reached statistical significance (Fisher’s exact test *P-*value < 0.05) (Supplementary Tables 2, 3). There were several possible reasons. First, there might be some differences in the frequency of the GZ allele among different ethnicities, and was relatively lower in the Chinese population. Under this condition, a larger cohort size might be needed to replicate our result further. Second, most of the studies mentioned above were based on the diagnosis of Parkinsonism. However, in our study, all patients were diagnosed with clinically established PD or probable PD. We proposed that other secondary Parkinsonism might be with the higher frequency of the GZ allele, but this proposal still needs further exploration studies on the secondary parkinsonism cohorts. Interestingly, two patients (PD-1 and PD-2) with premutation of *FMR1* CGG repeat expansion manifested depression, whereas only two of 11 patients harboring gray zone expansion repeats had depression symptoms. It is likely to be a tendency that patients with premutation of *FMR1* CGG repeat expansion were prone to be depressive. However, the samples were too small to ascertain this correlation, which needs larger samples to explore the relationship between depression and repeat size of *FMR1* CGG repeat expansion in the future.

However, there are still some limitations to this study. First, patients with repeat expansions were partially lost to follow-up, so the traceable follow-up data were not detailed enough. Therefore, we did not further explore the correlation between *FMR1* CGG repeat expansion and the disease progression of PD. Second, we only explored the correlation between *FMR1* CGG repeat expansions and PD without further exploration of the potential pathogenesis underlying it. Third, we collected DNA from peripheral blood cells rather than other tissues, such as the brain. DNA from different tissues may help study the correlation between FMR1 CGG repeat allele and PD, which needs to be further explored.

In conclusion, our study suggested that the premutation of *FMR1* CGG repeat expansions was related to PD in the Chinese population. However, the GZ allele of *FMR1* CGG repeat expansions was not significantly associated with PD in the Chinese population. Further studies with larger sample sizes and experimental studies are warranted to determine further the role of premature and GZ allele of *FMR1* CGG repeat expansions in PD.

## Data availability statement

The original contributions presented in this study are included in this article/Supplementary material, further inquiries can be directed to the corresponding authors.

## Ethics statement

The studies involving human participants were reviewed and approved by the Ethics Committee of Xiangya Hospital (Central South University). The patients/participants provided their written informed consent to participate in this study. Written informed consent was obtained from the individual(s) for the publication of any potentially identifiable images or data included in this article.

## Author contributions

JC and YZ: design, execution, and writing—original draft. XuZ, JX, QXi, HP, XiZ, and YX: methodology. JiaL and LZ: imaging materials. ZZ, YY, QXu, QS, and XY: participants enrollment and clinical data collection. JT and JinL: methodology and writing—review. JG: participants enrollment. BT: conception and participants enrollment. RD: blood samples collection and DNA extraction. QY and ZL: design, conception, writing—review and editing, and supervision. All authors contributed to the article and approved the submitted version.

## Footnotes

1
http://pd-mdcnc.com
