# Supplementary material for: Evaluation of the role of FMR1 CGG repeat allele in Parkinson’s disease from the Chinese population
Source: Front Aging Neurosci. 2023 Jul 31;15:1234027. doi: 10.3389/fnagi.2023.1234027 (PMC10423993; doi:10.3389/fnagi.2023.1234027)
Supplement: Supplementary file 1 [file Data_Sheet_1.PDF]

Supplementary Table 1. Analysis of *FMR1* gene gray-zone (GZ) allele burden in Parkinson's disease (45-54 repeats).

Group	Cases	Controls	*P*-Value (fisher)	OR (95% CI)
All cohort	11/2349	5/1067	>0.999	0.99 (0.31-3.68)
Female cohort	6/1112	3/548	>0.999	0.99 (0.21-6.11)
Male cohort	5/1237	2/519	>0.999	1.05 (0.17-11.05)
EOPD cohort	8/1108	5/1067	0.581	1.54 (0.44-6.01)
LOPD cohort	3/1241	5/1067	0.483	0.52 (0.08-2.66)

EOPD: early onset of Parkinson's disease; LOPD: late onset of Parkinson's disease.

Supplementary Table 2. Analysis of *FMR1* gene gray-zone (GZ) allele burden in Parkinson's disease (40-54 repeats)

Group	Cases	Controls	*P*-Value (fisher)	OR (95% CI)
All cohort	73/2287	24/1048	0.183	1.39 (0.86-2.33)
Female cohort	42/1076	15/536	0.317	1.39 (0.75-2.73)
Male cohort	31/1211	9/512	0.383	1.46 (0.67-3.50)
EOPD cohort	39/1077	24/1048	0.096	1.58 (0.92-2.77)
LOPD cohort	34/1210	24/1048	0.506	1.23 (0.70-2.18)

EOPD: early onset of Parkinson's disease; LOPD: late onset of Parkinson's disease.

Supplementary Table 3. Analysis of *FMR1* gene gray-zone (GZ) allele burden in Parkinson's disease (41-54 repeats)

Group	Cases	Controls	*P*-Value (fisher)	OR (95% CI)
All cohort	56/2304	22/1050	0.622	1.16 (0.69-2.01)
Female cohort	29/1089	14/537	>0.999	1.02 (0.52-2.11)
Male cohort	27/1215	8/513	0.457	1.42 (0.62-3.65)
EOPD cohort	30/1086	22/1050	0.400	1.32 (0.73-2.42)
LOPD cohort	26/1218	22/1050	>0.999	1.02 (0.55-1.90)

EOPD: early onset of Parkinson's disease; LOPD: late onset of Parkinson's disease.
